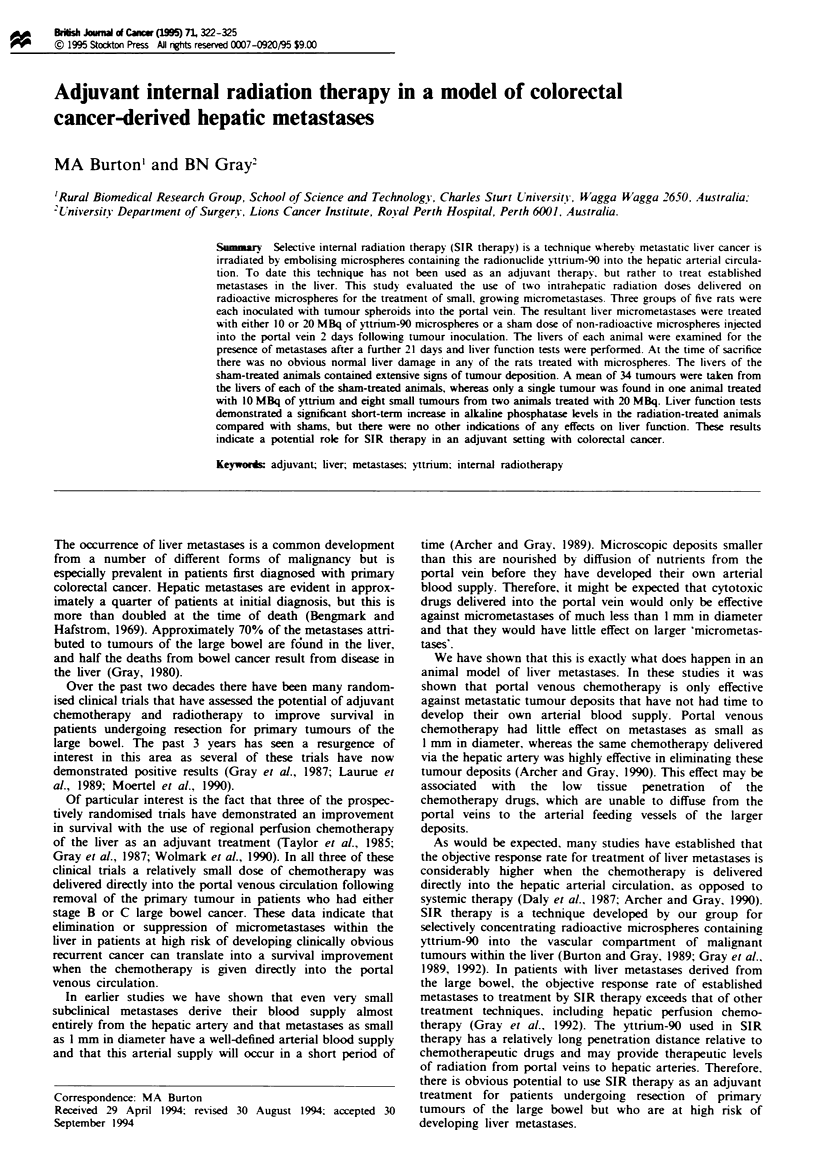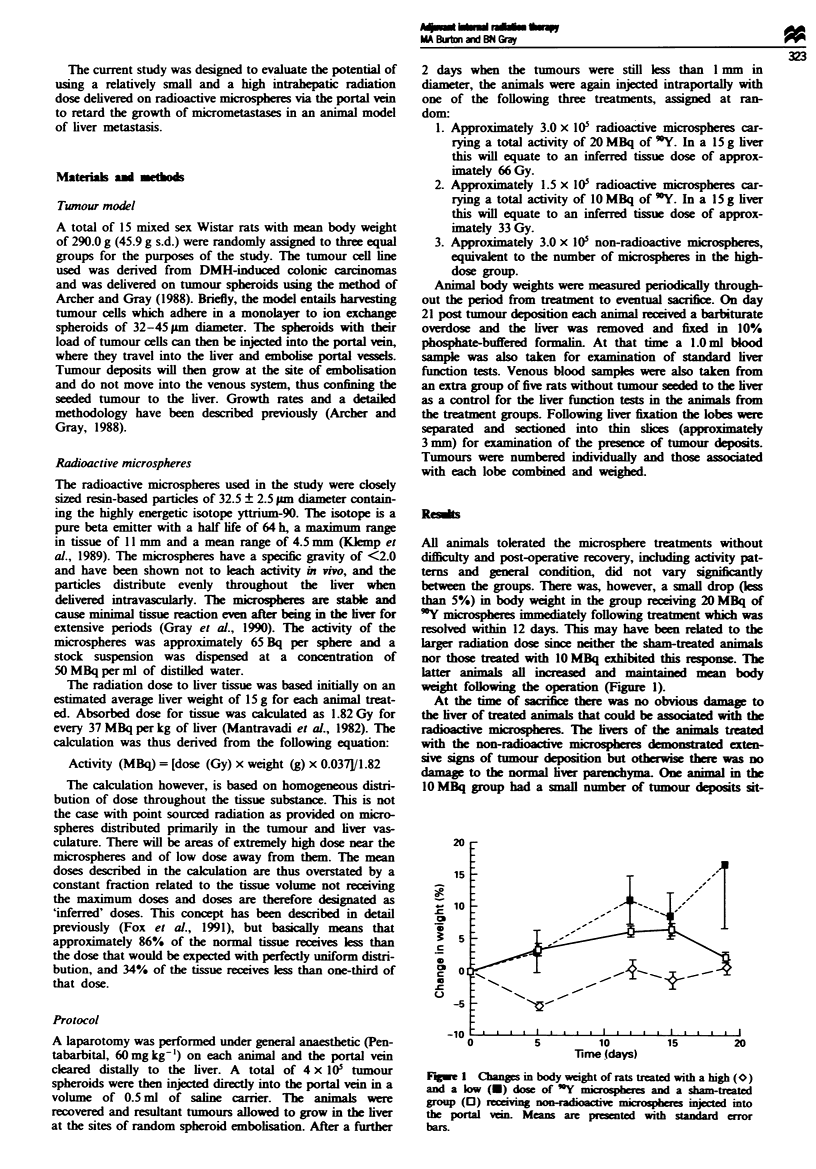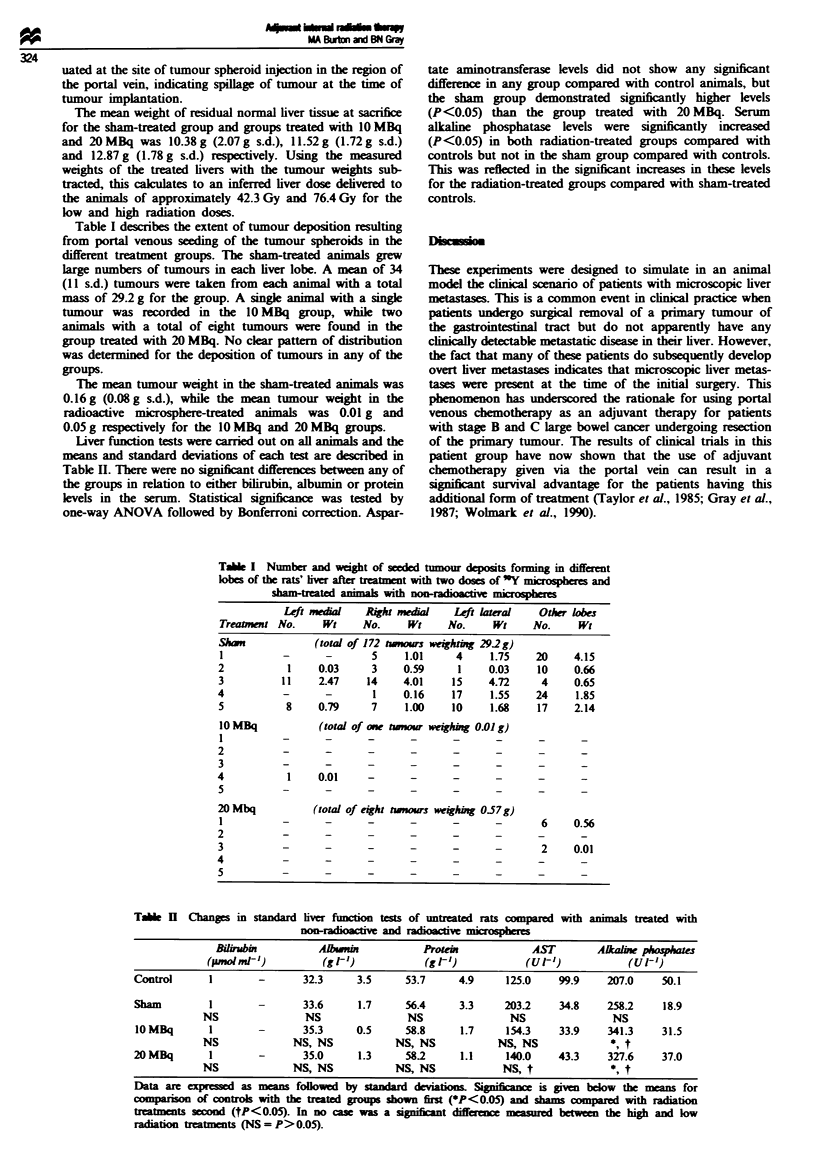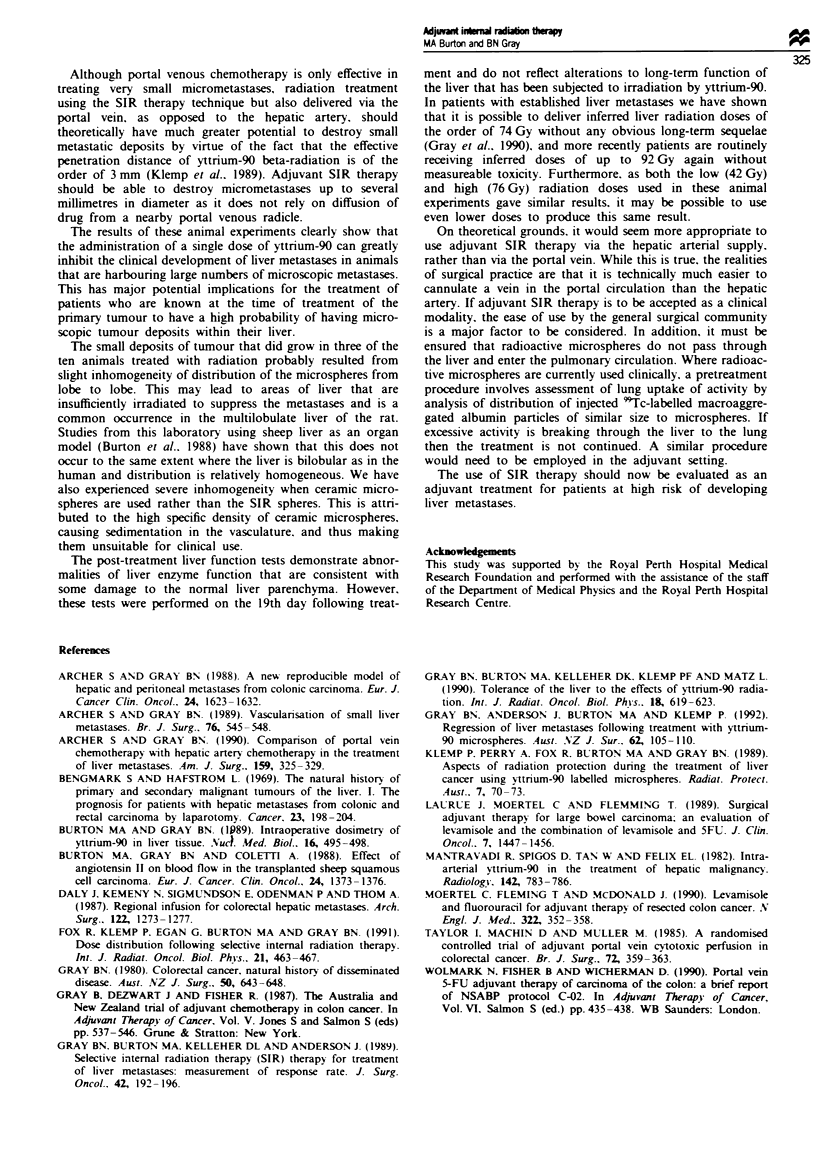# Adjuvant internal radiation therapy in a model of colorectal cancer-derived hepatic metastases.

**DOI:** 10.1038/bjc.1995.64

**Published:** 1995-02

**Authors:** M. A. Burton, B. N. Gray

**Affiliations:** School of Science and Technology, Charles Sturt University, Wagga Wagga, Australia.

## Abstract

Selective internal radiation therapy (SIR therapy) is a technique whereby metastatic liver cancer is irradiated by embolising microspheres containing the radionuclide yttrium-90 into the hepatic arterial circulation. To date this technique has not been used as an adjuvant therapy, but rather to treat established metastases in the liver. This study evaluated the use of two intrahepatic radiation doses delivered on radioactive microspheres for the treatment of small, growing micrometastases. Three groups of five rats were each inoculated with tumour spheroids into the portal vein. The resultant liver micrometastases were treated with either 10 or 20 MBq of yttrium-90 microspheres or a sham dose of non-radioactive microspheres injected into the portal vein 2 days following tumour inoculation. The livers of each animal were examined for the presence of metastases after a further 21 days and liver function tests were performed. At the time of sacrifice there was no obvious normal liver damage in any of the rats treated with microspheres. The livers of the sham-treated animals contained extensive signs of tumour deposition. A mean of 34 tumours were taken from the livers of each of the sham-treated animals, whereas only a single tumour was found in one animal treated with 10 MBq of yttrium and eight small tumours from two animals treated with 20 MBq. Liver function tests demonstrated a significant short-term increase in alkaline phosphatase levels in the radiation-treated animals compared with shams, but there were no other indications of any effects on liver function. These results indicate a potential role for SIR therapy in an adjuvant setting with colorectal cancer.


British Journal of Cancer (1995) 71, 322-325

© 1995 Stockton Press All rights reserved 0007-0920/95 $9.00

### Adjuvant internal radiation therapy in a model of colorectal cancer-derived hepatic metastases

MA Burton[1] and BN Gray[2]

[1]*Rural Biomedical Research Group, School of Science and Technology, Charles Sturt University, Wagga Wagga 2650, Australia;* [2]*University Department of Surgery, Lions Cancer Institute, Royal Perth Hospital, Perth 6001, Australia.*

**Summary** Selective internal radiation therapy (SIR therapy) is a technique whereby metastatic liver cancer is irradiated by embolising microspheres containing the radionuclide yttrium-90 into the hepatic arterial circulation. To date this technique has not been used as an adjuvant therapy, but rather to treat established metastases in the liver. This study evaluated the use of two intrahepatic radiation doses delivered on radioactive microspheres for the treatment of small, growing micrometastases. Three groups of five rats were each inoculated with tumour spheroids into the portal vein. The resultant liver micrometastases were treated with either 10 or 20 MBq of yttrium-90 microspheres or a sham dose of non-radioactive microspheres injected into the portal vein 2 days following tumour inoculation. The livers of each animal were examined for the presence of metastases after a further 21 days and liver function tests were performed. At the time of sacrifice there was no obvious normal liver damage in any of the rats treated with microspheres. The livers of the sham-treated animals contained extensive signs of tumour deposition. A mean of 34 tumours were taken from the livers of each of the sham-treated animals, whereas only a single tumour was found in one animal treated with 10 MBq of yttrium and eight small tumours from two animals treated with 20 MBq. Liver function tests demonstrated a significant short-term increase in alkaline phosphatase levels in the radiation-treated animals compared with shams, but there were no other indications of any effects on liver function. These results indicate a potential role for SIR therapy in an adjuvant setting with colorectal cancer.

**Keywords:** adjuvant; liver; metastases; yttrium; internal radiotherapy

The occurrence of liver metastases is a common development from a number of different forms of malignancy but is especially prevalent in patients first diagnosed with primary colorectal cancer. Hepatic metastases are evident in approximately a quarter of patients at initial diagnosis, but this is more than doubled at the time of death (Bengmark and Hafstrom, 1969). Approximately 70% of the metastases attributed to tumours of the large bowel are found in the liver, and half the deaths from bowel cancer result from disease in the liver (Gray, 1980).

Over the past two decades there have been many randomised clinical trials that have assessed the potential of adjuvant chemotherapy and radiotherapy to improve survival in patients undergoing resection for primary tumours of the large bowel. The past 3 years has seen a resurgence of interest in this area as several of these trials have now demonstrated positive results (Gray *et al.*, 1987; Laurue *et al.*, 1989; Moertel *et al.*, 1990).

Of particular interest is the fact that three of the prospectively randomised trials have demonstrated an improvement in survival with the use of regional perfusion chemotherapy of the liver as an adjuvant treatment (Taylor *et al.*, 1985; Gray *et al.*, 1987; Wolmark *et al.*, 1990). In all three of these clinical trials a relatively small dose of chemotherapy was delivered directly into the portal venous circulation following removal of the primary tumour in patients who had either stage B or C large bowel cancer. These data indicate that elimination or suppression of micrometastases within the liver in patients at high risk of developing clinically obvious recurrent cancer can translate into a survival improvement when the chemotherapy is given directly into the portal venous circulation.

In earlier studies we have shown that even very small subclinical metastases derive their blood supply almost entirely from the hepatic artery and that metastases as small as 1 mm in diameter have a well-defined arterial blood supply and that this arterial supply will occur in a short period of time (Archer and Gray, 1989). Microscopic deposits smaller than this are nourished by diffusion of nutrients from the portal vein before they have developed their own arterial blood supply. Therefore, it might be expected that cytotoxic drugs delivered into the portal vein would only be effective against micrometastases of much less than 1 mm in diameter and that they would have little effect on larger 'micrometastases'.

We have shown that this is exactly what does happen in an animal model of liver metastases. In these studies it was shown that portal venous chemotherapy is only effective against metastatic tumour deposits that have not had time to develop their own arterial blood supply. Portal venous chemotherapy had little effect on metastases as small as 1 mm in diameter, whereas the same chemotherapy delivered via the hepatic artery was highly effective in eliminating these tumour deposits (Archer and Gray, 1990). This effect may be associated with the low tissue penetration of the chemotherapy drugs, which are unable to diffuse from the portal veins to the arterial feeding vessels of the larger deposits.

As would be expected, many studies have established that the objective response rate for treatment of liver metastases is considerably higher when the chemotherapy is delivered directly into the hepatic arterial circulation, as opposed to systemic therapy (Daly *et al.*, 1987; Archer and Gray, 1990). SIR therapy is a technique developed by our group for selectively concentrating radioactive microspheres containing yttrium-90 into the vascular compartment of malignant tumours within the liver (Burton and Gray, 1989; Gray *et al.*, 1989, 1992). In patients with liver metastases derived from the large bowel, the objective response rate of established metastases to treatment by SIR therapy exceeds that of other treatment techniques, including hepatic perfusion chemotherapy (Gray *et al.*, 1992). The yttrium-90 used in SIR therapy has a relatively long penetration distance relative to chemotherapeutic drugs and may provide therapeutic levels of radiation from portal veins to hepatic arteries. Therefore, there is obvious potential to use SIR therapy as an adjuvant treatment for patients undergoing resection of primary tumours of the large bowel but who are at high risk of developing liver metastases.

Correspondence: MA Burton
Received 29 April 1994; revised 30 August 1994; accepted 30 September 1994

The current study was designed to evaluate the potential of using a relatively small and a high intrahepatic radiation dose delivered on radioactive microspheres via the portal vein to retard the growth of micrometastases in an animal model of liver metastasis.

### Materials and methods

### *Tumour model*

A total of 15 mixed sex Wistar rats with mean body weight of 290.0 g (45.9 g s.d.) were randomly assigned to three equal groups for the purposes of the study. The tumour cell line used was derived from DMH-induced colonic carcinomas and was delivered on tumour spheroids using the method of Archer and Gray (1988). Briefly, the model entails harvesting tumour cells which adhere in a monolayer to ion exchange spheroids of 32–45 μm diameter. The spheroids with their load of tumour cells can then be injected into the portal vein, where they travel into the liver and embolise portal vessels. Tumour deposits will then grow at the site of embolisation and do not move into the venous system, thus confining the seeded tumour to the liver. Growth rates and a detailed methodology have been described previously (Archer and Gray, 1988).

### *Radioactive microspheres*

The radioactive microspheres used in the study were closely sized resin-based particles of 32.5 ± 2.5 μm diameter containing the highly energetic isotope yttrium-90. The isotope is a pure beta emitter with a half life of 64 h, a maximum range in tissue of 11 mm and a mean range of 4.5 mm (Klemp *et al.*, 1989). The microspheres have a specific gravity of <2.0 and have been shown not to leach activity *in vivo*, and the particles distribute evenly throughout the liver when delivered intravascularly. The microspheres are stable and cause minimal tissue reaction even after being in the liver for extensive periods (Gray *et al.*, 1990). The activity of the microspheres was approximately 65 Bq per sphere and a stock suspension was dispensed at a concentration of 50 MBq per ml of distilled water.

The radiation dose to liver tissue was based initially on an estimated average liver weight of 15 g for each animal treated. Absorbed dose for tissue was calculated as 1.82 Gy for every 37 MBq per kg of liver (Mantravadi *et al.*, 1982). The calculation was thus derived from the following equation:

$$\text{Activity (MBq)} = [\text{dose (Gy)} \times \text{weight (g)} \times 0.037]/1.82$$

The calculation however, is based on homogeneous distribution of dose throughout the tissue substance. This is not the case with point sourced radiation as provided on microspheres distributed primarily in the tumour and liver vasculature. There will be areas of extremely high dose near the microspheres and of low dose away from them. The mean doses described in the calculation are thus overstated by a constant fraction related to the tissue volume not receiving the maximum doses and doses are therefore designated as 'inferred' doses. This concept has been described in detail previously (Fox *et al.*, 1991), but basically means that approximately 86% of the normal tissue receives less than the dose that would be expected with perfectly uniform distribution, and 34% of the tissue receives less than one-third of that dose.

### *Protocol*

A laparotomy was performed under general anaesthetic (Pentabarbital, 60 mg kg$^{-1}$) on each animal and the portal vein cleared distally to the liver. A total of $4 \times 10^5$ tumour spheroids were then injected directly into the portal vein in a volume of 0.5 ml of saline carrier. The animals were recovered and resultant tumours allowed to grow in the liver at the sites of random spheroid embolisation. After a further 2 days when the tumours were still less than 1 mm in diameter, the animals were again injected intraportally with one of the following three treatments, assigned at random:

1. Approximately $3.0 \times 10^5$ radioactive microspheres carrying a total activity of 20 MBq of $^{90}$Y. In a 15 g liver this will equate to an inferred tissue dose of approximately 66 Gy.
2. Approximately $1.5 \times 10^5$ radioactive microspheres carrying a total activity of 10 MBq of $^{90}$Y. In a 15 g liver this will equate to an inferred tissue dose of approximately 33 Gy.
3. Approximately $3.0 \times 10^5$ non-radioactive microspheres, equivalent to the number of microspheres in the high-dose group.

Animal body weights were measured periodically throughout the period from treatment to eventual sacrifice. On day 21 post tumour deposition each animal received a barbiturate overdose and the liver was removed and fixed in 10% phosphate-buffered formalin. At that time a 1.0 ml blood sample was also taken for examination of standard liver function tests. Venous blood samples were also taken from an extra group of five rats without tumour seeded to the liver as a control for the liver function tests in the animals from the treatment groups. Following liver fixation the lobes were separated and sectioned into thin slices (approximately 3 mm) for examination of the presence of tumour deposits. Tumours were numbered individually and those associated with each lobe combined and weighed.

### Results

All animals tolerated the microsphere treatments without difficulty and post-operative recovery, including activity patterns and general condition, did not vary significantly between the groups. There was, however, a small drop (less than 5%) in body weight in the group receiving 20 MBq of $^{90}$Y microspheres immediately following treatment which was resolved within 12 days. This may have been related to the larger radiation dose since neither the sham-treated animals nor those treated with 10 MBq exhibited this response. The latter animals all increased and maintained mean body weight following the operation (Figure 1).

At the time of sacrifice there was no obvious damage to the liver of treated animals that could be associated with the radioactive microspheres. The livers of the animals treated with the non-radioactive microspheres demonstrated extensive signs of tumour deposition but otherwise there was no damage to the normal liver parenchyma. One animal in the 10 MBq group had a small number of tumour deposits sit-

**Figure 1** Changes in body weight of rats treated with a high (◇) and a low (■) dose of $^{90}$Y microspheres and a sham-treated group (□) receiving non-radioactive microspheres injected into the portal vein. Means are presented with standard error bars.

uated at the site of tumour spheroid injection in the region of the portal vein, indicating spillage of tumour at the time of tumour implantation.

The mean weight of residual normal liver tissue at sacrifice for the sham-treated group and groups treated with 10 MBq and 20 MBq was 10.38 g (2.07 g s.d.), 11.52 g (1.72 g s.d.) and 12.87 g (1.78 g s.d.) respectively. Using the measured weights of the treated livers with the tumour weights subtracted, this calculates to an inferred liver dose delivered to the animals of approximately 42.3 Gy and 76.4 Gy for the low and high radiation doses.

Table I describes the extent of tumour deposition resulting from portal venous seeding of the tumour spheroids in the different treatment groups. The sham-treated animals grew large numbers of tumours in each liver lobe. A mean of 34 (11 s.d.) tumours were taken from each animal with a total mass of 29.2 g for the group. A single animal with a single tumour was recorded in the 10 MBq group, while two animals with a total of eight tumours were found in the group treated with 20 MBq. No clear pattern of distribution was determined for the deposition of tumours in any of the groups.

The mean tumour weight in the sham-treated animals was 0.16 g (0.08 g s.d.), while the mean tumour weight in the radioactive microsphere-treated animals was 0.01 g and 0.05 g respectively for the 10 MBq and 20 MBq groups.

Liver function tests were carried out on all animals and the means and standard deviations of each test are described in Table II. There were no significant differences between any of the groups in relation to either bilirubin, albumin or protein levels in the serum. Statistical significance was tested by one-way ANOVA followed by Bonferroni correction. Aspartate aminotransferase levels did not show any significant difference in any group compared with control animals, but the sham group demonstrated significantly higher levels ($P<0.05$) than the group treated with 20 MBq. Serum alkaline phosphatase levels were significantly increased ($P<0.05$) in both radiation-treated groups compared with controls but not in the sham group compared with controls. This was reflected in the significant increases in these levels for the radiation-treated groups compared with sham-treated controls.

### Discussion

These experiments were designed to simulate in an animal model the clinical scenario of patients with microscopic liver metastases. This is a common event in clinical practice when patients undergo surgical removal of a primary tumour of the gastrointestinal tract but do not apparently have any clinically detectable metastatic disease in their liver. However, the fact that many of these patients do subsequently develop overt liver metastases indicates that microscopic liver metastases were present at the time of the initial surgery. This phenomenon has underscored the rationale for using portal venous chemotherapy as an adjuvant therapy for patients with stage B and C large bowel cancer undergoing resection of the primary tumour. The results of clinical trials in this patient group have now shown that the use of adjuvant chemotherapy given via the portal vein can result in a significant survival advantage for the patients having this additional form of treatment (Taylor *et al.*, 1985; Gray *et al.*, 1987; Wolmark *et al.*, 1990).

**Table I** Number and weight of seeded tumour deposits forming in different lobes of the rats' liver after treatment with two doses of $^{90}Y$ microspheres and sham-treated animals with non-radioactive microspheres

| | *Left medial* | | *Right medial* | | *Left lateral* | | *Other lobes* | |
|---|---|---|---|---|---|---|---|---|
| *Treatment* | *No.* | *Wt* | *No.* | *Wt* | *No.* | *Wt* | *No.* | *Wt* |
| *Sham* | *(total of 172 tumours weighting 29.2 g)* | | | | | | | |
| 1 | – | – | 5 | 1.01 | 4 | 1.75 | 20 | 4.15 |
| 2 | 1 | 0.03 | 3 | 0.59 | 1 | 0.03 | 10 | 0.66 |
| 3 | 11 | 2.47 | 14 | 4.01 | 15 | 4.72 | 4 | 0.65 |
| 4 | – | – | 1 | 0.16 | 17 | 1.55 | 24 | 1.85 |
| 5 | 8 | 0.79 | 7 | 1.00 | 10 | 1.68 | 17 | 2.14 |
| 10 MBq | *(total of one tumour weighing 0.01 g)* | | | | | | | |
| 1 | – | – | – | – | – | – | – | – |
| 2 | – | – | – | – | – | – | – | – |
| 3 | – | – | – | – | – | – | – | – |
| 4 | 1 | 0.01 | – | – | – | – | – | – |
| 5 | – | – | – | – | – | – | – | – |
| 20 Mbq | *(total of eight tumours weighing 0.57 g)* | | | | | | | |
| 1 | – | – | – | – | – | – | 6 | 0.56 |
| 2 | – | – | – | – | – | – | – | – |
| 3 | – | – | – | – | – | – | 2 | 0.01 |
| 4 | – | – | – | – | – | – | – | – |
| 5 | – | – | – | – | – | – | – | – |

**Table II** Changes in standard liver function tests of untreated rats compared with animals treated with non-radioactive and radioactive microspheres

| | *Bilirubin* ($\mu mol\ ml^{-1}$) | | *Albumin* ($g\ l^{-1}$) | | *Protein* ($g\ l^{-1}$) | | *AST* ($U\ l^{-1}$) | | *Alkaline phosphates* ($U\ l^{-1}$) | |
|---|---|---|---|---|---|---|---|---|---|---|
| Control | 1 | – | 32.3 | 3.5 | 53.7 | 4.9 | 125.0 | 99.9 | 207.0 | 50.1 |
| Sham | 1 | – | 33.6 | 1.7 | 56.4 | 3.3 | 203.2 | 34.8 | 258.2 | 18.9 |
| | NS | | NS | | NS | | NS | | NS | |
| 10 MBq | 1 | – | 35.3 | 0.5 | 58.8 | 1.7 | 154.3 | 33.9 | 341.3 | 31.5 |
| | NS | | NS, NS | | NS, NS | | NS, NS | | *, † | |
| 20 MBq | 1 | – | 35.0 | 1.3 | 58.2 | 1.1 | 140.0 | 43.3 | 327.6 | 37.0 |
| | NS | | NS, NS | | NS, NS | | NS, † | | *, † | |

Data are expressed as means followed by standard deviations. Significance is given below the means for comparison of controls with the treated groups shown first (*$P<0.05$) and shams compared with radiation treatments second (†$P<0.05$). In no case was a significant difference measured between the high and low radiation treatments (NS = $P>0.05$).

Although portal venous chemotherapy is only effective in treating very small micrometastases, radiation treatment using the SIR therapy technique but also delivered via the portal vein, as opposed to the hepatic artery, should theoretically have much greater potential to destroy small metastatic deposits by virtue of the fact that the effective penetration distance of yttrium-90 beta-radiation is of the order of 3 mm (Klemp *et al.*, 1989). Adjuvant SIR therapy should be able to destroy micrometastases up to several millimetres in diameter as it does not rely on diffusion of drug from a nearby portal venous radicle.

The results of these animal experiments clearly show that the administration of a single dose of yttrium-90 can greatly inhibit the clinical development of liver metastases in animals that are harbouring large numbers of microscopic metastases. This has major potential implications for the treatment of patients who are known at the time of treatment of the primary tumour to have a high probability of having microscopic tumour deposits within their liver.

The small deposits of tumour that did grow in three of the ten animals treated with radiation probably resulted from slight inhomogeneity of distribution of the microspheres from lobe to lobe. This may lead to areas of liver that are insufficiently irradiated to suppress the metastases and is a common occurrence in the multilobulate liver of the rat. Studies from this laboratory using sheep liver as an organ model (Burton *et al.*, 1988) have shown that this does not occur to the same extent where the liver is bilobular as in the human and distribution is relatively homogeneous. We have also experienced severe inhomogeneity when ceramic microspheres are used rather than the SIR spheres. This is attributed to the high specific density of ceramic microspheres, causing sedimentation in the vasculature, and thus making them unsuitable for clinical use.

The post-treatment liver function tests demonstrate abnormalities of liver enzyme function that are consistent with some damage to the normal liver parenchyma. However, these tests were performed on the 19th day following treatment and do not reflect alterations to long-term function of the liver that has been subjected to irradiation by yttrium-90. In patients with established liver metastases we have shown that it is possible to deliver inferred liver radiation doses of the order of 74 Gy without any obvious long-term sequelae (Gray *et al.*, 1990), and more recently patients are routinely receiving inferred doses of up to 92 Gy again without measureable toxicity. Furthermore, as both the low (42 Gy) and high (76 Gy) radiation doses used in these animal experiments gave similar results, it may be possible to use even lower doses to produce this same result.

On theoretical grounds, it would seem more appropriate to use adjuvant SIR therapy via the hepatic arterial supply, rather than via the portal vein. While this is true, the realities of surgical practice are that it is technically much easier to cannulate a vein in the portal circulation than the hepatic artery. If adjuvant SIR therapy is to be accepted as a clinical modality, the ease of use by the general surgical community is a major factor to be considered. In addition, it must be ensured that radioactive microspheres do not pass through the liver and enter the pulmonary circulation. Where radioactive microspheres are currently used clinically, a pretreatment procedure involves assessment of lung uptake of activity by analysis of distribution of injected $^{99}$Tc-labelled macroaggregated albumin particles of similar size to microspheres. If excessive activity is breaking through the liver to the lung then the treatment is not continued. A similar procedure would need to be employed in the adjuvant setting.

The use of SIR therapy should now be evaluated as an adjuvant treatment for patients at high risk of developing liver metastases.

**Acknowledgements**

This study was supported by the Royal Perth Hospital Medical Research Foundation and performed with the assistance of the staff of the Department of Medical Physics and the Royal Perth Hospital Research Centre.

**References**

ARCHER S AND GRAY BN (1988). A new reproducible model of hepatic and peritoneal metastases from colonic carcinoma. *Eur. J. Cancer Clin. Oncol.*, **24**, 1623–1632.

ARCHER S AND GRAY BN (1989). Vascularisation of small liver metastases. *Br. J. Surg.*, **76**, 545–548.

ARCHER S AND GRAY BN. (1990). Comparison of portal vein chemotherapy with hepatic artery chemotherapy in the treatment of liver metastases. *Am. J. Surg.*, **159**, 325–329.

BENGMARK S AND HAFSTROM L. (1969). The natural history of primary and secondary malignant tumours of the liver. I. The prognosis for patients with hepatic metastases from colonic and rectal carcinoma by laparotomy. *Cancer*, **23**, 198–204.

BURTON MA AND GRAY BN. (1989). Intraoperative dosimetry of yttrium-90 in liver tissue. *Nucl. Med. Biol.*, **16**, 495–498.

BURTON MA, GRAY BN AND COLETTI A. (1988). Effect of angiotensin II on blood flow in the transplanted sheep squamous cell carcinoma. *Eur. J. Cancer. Clin. Oncol.*, **24**, 1373–1376.

DALY J, KEMENY N, SIGMUNDSON E, ODENMAN P AND THOM A. (1987). Regional infusion for colorectal hepatic metastases. *Arch. Surg.*, **122**, 1273–1277.

FOX R, KLEMP P, EGAN G, BURTON MA AND GRAY BN. (1991). Dose distribution following selective internal radiation therapy. *Int. J. Radiat. Oncol. Biol. Phys.*, **21**, 463–467.

GRAY BN. (1980). Colorectal cancer, natural history of disseminated disease. *Aust. NZ J. Surg.*, **50**, 643–648.

GRAY B, DEZWART J AND FISHER R. (1987). The Australia and New Zealand trial of adjuvant chemotherapy in colon cancer. In *Adjuvant Therapy of Cancer*, Vol. V. Jones S and Salmon S (eds) pp. 537–546. Grune & Stratton: New York.

GRAY BN, BURTON MA, KELLEHER DL AND ANDERSON J. (1989). Selective internal radiation therapy (SIR) therapy for treatment of liver metastases: measurement of response rate. *J. Surg. Oncol.*, **42**, 192–196.

GRAY BN, BURTON MA, KELLEHER DK, KLEMP PF AND MATZ L. (1990). Tolerance of the liver to the effects of yttrium-90 radiation. *Int. J. Radiat. Oncol. Biol. Phys.*, **18**, 619–623.

GRAY BN, ANDERSON J, BURTON MA AND KLEMP P. (1992). Regression of liver metastases following treatment with yttrium-90 microspheres. *Aust. NZ J. Sur.*, **62**, 105–110.

KLEMP P, PERRY A, FOX R, BURTON MA AND GRAY BN. (1989). Aspects of radiation protection during the treatment of liver cancer using yttrium-90 labelled microspheres. *Radiat. Protect. Aust.*, **7**, 70–73.

LAURUE J, MOERTEL C AND FLEMMING T. (1989). Surgical adjuvant therapy for large bowel carcinoma: an evaluation of levamisole and the combination of levamisole and 5FU. *J. Clin. Oncol.*, **7**, 1447–1456.

MANTRAVADI R, SPIGOS D, TAN W AND FELIX EL. (1982). Intra-arterial yttrium-90 in the treatment of hepatic malignancy. *Radiology*, **142**, 783–786.

MOERTEL C, FLEMING T AND McDONALD J. (1990). Levamisole and fluorouracil for adjuvant therapy of resected colon cancer. *N Engl. J. Med.*, **322**, 352–358.

TAYLOR I, MACHIN D AND MULLER M. (1985). A randomised controlled trial of adjuvant portal vein cytotoxic perfusion in colorectal cancer. *Br. J. Surg.*, **72**, 359–363.

WOLMARK N, FISHER B AND WICHERMAN D. (1990). Portal vein 5-FU adjuvant therapy of carcinoma of the colon: a brief report of NSABP protocol C-02. In *Adjuvant Therapy of Cancer*, Vol. VI, Salmon S (ed.) pp. 435–438. WB Saunders: London.